# The relationship between peer victimization and depression: a moderated mediation model of self-esteem and resilience

**DOI:** 10.3389/fpsyg.2026.1829714

**Published:** 2026-05-25

**Authors:** Pingyan Zhou, Jiani Peng, Cai Zhang, Zhe Wang, Jian Liu

**Affiliations:** 1 School of Psychology, Qufu Normal University, Qufu, China; 2 Collaborative Innovation Center of Assessment for Basic Education Quality, Beijing Normal University, Beijing, China

**Keywords:** depression, moderated mediation model, peer victimization, resilience, self-esteem

## Abstract

**Introduction:**

Although peer victimization is a known risk factor for depression, the specific mediating and moderating processes need further study.

**Methods:**

This research used self-report data from 58,753 fourth graders to measure peer victimization, selfesteem, resilience, and depression.

**Results:**

The analysis showed that peer victimization was associated with depression both directly (71.92%) and indirectly (28.08%). The relationship between peer victimization and depression was partially mediated by self-esteem. Resilience exhibited a protective-enhancing effect in the relationship between peer victimization and depression: its role strengthened as peer victimization worsens. Whereas, resilience demonstrated protective-reactive effect of both peer victimization on self-esteem and self-esteem on depression, its protective effect attenuated when high victimization coincided with very low self-esteem. Clarified that protective-enhancing applies only to the direct path, while protective-reactive applies to the two indirect paths.

**Conclusion:**

Although peer victimization is a crucial predictor of depression, effective interventions for depression must extend beyond fostering supportive peer environments by also targeting key internal processes—specifically, by building resilience and disrupting the internalization of victimization into negative self-esteem.

## Introduction

Peer victimization—defined as repeated, intentional negative actions by one or more students against a child (Olweus, 1993)—manifests in physical, verbal, relational, and exclusionary forms (Crick and Bigbee, 1998; Wang et al., 2009). Up to 30.4% of children worldwide experience such bullying during their formative years (Steggerda et al., 2024; Xu et al., 2020). Individuals involved in bullying typically falling into four primary roles: the victim, the bully, the bully-victim, and the non-involved (Sigurdson et al., 2015). There is convincing evidence suggesting that peer victimization has seriously negative consequences on students’ physical and mental health (Brendgen et al., 2019), such as academic failure (Guo et al., 2024), addictive behavior (Chen et al., 2023), self-injury (Li et al., 2024), low self-esteem, anxiety, depression (Perret et al., 2021), even contributing to suicid (Koyanagi et al., 2019). Ample studies have found that depression is seen as a permanent accomplice of peer victimization (Huang et al., 2025; Sigurdson et al., 2015). Meta-analysis of longitudinal studies demonstrated a two-fold increased risk of developing depression in both victims and perpetrators of bullying compared to their non-involved peers (Ttofi et al., 2011). Yet, the underlying mediating and moderating mechanisms are largely unknown, despite being key to understanding the etiology and designing effective interventions. Hence, this study aimed to explore the mediating role of self-esteem in the relationship between peer victimization and depression, and the moderating effect of resilience on this mediating pathway.

### Peer victimization and depression

Depression is characterized by intensified negative affect, which is often associated with a diminished sense of self-worth, reduced confidence, and an impaired capacity to overcome ongoing life challenges (Dervishi et al., 2019). Studies found that being either a victim or bully is prone to developing depression (Copeland et al., 2013; Klomek et al., 2013). The more serious the peer victimization, the more intensity of depressive symptoms (Abela, 2002). Studies have identified robust link between victimization frequency and depressive symptom severity, irrespective of gender, age, or academic performance (Dervishi et al., 2019; Ledley et al., 2006). Additionally, evidence has substantiated that peer victimization serves as a strong predictor of depression in children, and is further associated with severe depression and comorbid conditions in adolescents (Copeland et al., 2013; Klomek et al., 2013). According to a meta-analytic review spanning 20 years of research, peer victimization demonstrates the strongest link with depression when compared to other forms of maladjustment, such as anxiety (Hawker and Boulton, 2000). Depression is diagnosed in more than 50% of adolescents who died by suicide (Chiou et al., 2006). Further, the analysis revealed that peer victimization has direct and persistent effects, resulting in a significant increase in the likelihood of later depression and suicidality, even after accounting for other established suicide risk factors (Copeland et al., 2013; Kaltiala-Heino et al., 1999; Klomek et al., 2013; Ttofi et al., 2011), and the reason maybe that depression potentially hinders their development into independent adults (Copeland et al., 2013).

### The mediating effect of self-esteem

Self-esteem refers to one’s evaluation of self-worth and competence (Rosenberg, 1965). A robust body of evidence indicates a strong inverse relationship between self-esteem and peer victimization (Cetinkaya et al., 2009; Laftman and Modin, 2017) and low self-esteem is a well-established risk factor for depression, with effects twice as large as the reverse pathway (Aebi and Orth, 2025; Sowislo and Orth, 2013). Self-esteem is usually the inherent motivation or need for individuals to maintain and defend positive evaluations of self (Pyszczynski et al., 2004). Self-esteem, which solidified during childhood, represents the evaluative and affective dimension of the self-concept, which remains susceptible to diverse internal and external influences, especially during the transformative period of adolescence (Reitz, 2022). Cetinkaya et al. found that adolescents who have been exposed to bullying reported lower levels of self-esteem, refered to feelings of rejection, disconnection, and low self-regard, compared with control participants (Brito and Oliveira, 2013; Cetinkaya et al., 2009; Hawker and Boulton, 2000). Particularly, the vulnerability effect is so robust that its relationship remains stable when some important variables, such as gender, age, measurements are controlled.

According to the integrated theory of depression (Metalsky et al., 1993), peer victimization leads children to internalize bullying as self-blame, attributing negative events to internal, stable, and global causes. This process lowers self-esteem (Salmon et al., 1998; Tsaousis, 2016), which in turn increases vulnerability to depression (Abela, 2002). Namely, the effect of peer victimization on depression operates through the mechanism of self-esteem. Children who were frequently victimized demonstrates a pronounced tendency toward sustained low self-esteem, a factor directly associated with the development of depression both concurrently and later in life (Klomek et al., 2008). Furthermore, empirical evidence confirmed that self-esteem serves in a mediating role in the relationship linking exposure to major negative events to the development of depression (Luk et al., 2016), such as parental divorse (Palosaari et al., 1996), and social networking sites addiction (Wang et al., 2018). Drawing on the above review, we hypothesized that peer victimization is associated with depression through the pathway of self-esteem.

### The moderating effect of resilience

Although peer victimization can increase the risk of depression by damaging self-esteem (Luk et al., 2016), not all children who experienced peer victimization contributing to adverse outcomes, avoiding severe depression. Put differently, some individuals exhibit positive adaptation to peer victimization and remained free from both depressive symptom and suicidal ideation. This variation may originate from the protective factors (Luthar, 2006; Masten, 2001), which can reduce the negative effects of stressful events on depression, such as social support, resilience, coping style, self-evaluation, emotional regulation (Kliewer, 2016; Shaw et al., 2019), among which the role of resilience has received particularly attention in recent years (Troy et al., 2023). Resilience can be as a personal trait to mitigate the adverse effects of risk factors on individuals, and also refers to a dynamic phenomenon that although individuals have experienced serious adversities, they still have relatively good adaptation or development (Luthar et al., 2000; Rutter, 1985). The essence of resilience is that individuals have the healthier outcomes in facing extreme stress and adversity situations (Cohen et al., 2019; Losel and Bliesener, 1994), which demonstrates a multifaceted composition, such as emotion regulation, problem-solving skills, social support utilization, attentional control, psychological toughness, and self-efficacy (Connor and Davidson, 2003; Masten, 2018).

Studies indicated that resilience levels led to differential responses to similar adversities (Moore and Woodcock, 2017). For example, individuals with lower resilience are at a greater risk of becoming victims of school bullying (Hinduja and Patchin, 2017). Conversely, even after controlling for social support, higher resilience is linked to reduced levels of perceived stress (Jose and Novaco, 2016). Resilience has been conceptualized through different moderating models (Luthar et al., 2000). In protective-enhancing models, resilience buffers negative outcomes more strongly under high adversity; in protective-reactive models, the buffering effect weakens as adversity increases. These models guide our examination of how resilience moderates the pathways from peer victimization to depression via self-esteem. The organism-environment interaction model (Cummings et al., 2002) also states that individuals with specific personality traits respond differentially to similar environmental contexts. This theoretical framework suggests that the relationship between peer victimization and self-esteem is moderated by certain child characteristics such as resilience. Based on the protective-protective model of resilience (Fergus and Zimmerman, 2005), different protective resources, such as resilience and self-esteem, interact to create a stronger combined effect, contributing to better outcomes when facing adversity. Specifically, resilience can enhance the role of self-esteem in reducing depression. Some studies have already found that resilience could enhance the impact of self-esteem on mental disorder (Doyle and Catling, 2022). We therefore proposed that resilience predicts how peer victimization is associated with depression by shaping self-esteem.

### The current study

Drawing on the integrated theory of depression and the moderating models of resilience, this study focused on a moderated mediation model to clarify how (via self-esteem) and when (depending on resilience) peer victimization predicts depression. Based on the aforementioned literature, the aims of our study were twofold: (1) to explore whether self-esteem would mediate the association between peer victimization and depression; (2) to examine whether resilience would moderate the mediation process. We proposed the following hypotheses:

*Hypothesis 1*: Peer victimization would be negatively associated with self-esteem, which in turn would be strongly related to higher risk of depression.

*Hypothesis 2*: Resilience would moderate the direct and indirect pathways from peer victimization to depression.

## Method

### Participants

This study involved a total of 58,753 fourth-grade students (mean age = 10.83 ± 0.83 years) from 280 urban primary schools in Zhengzhou City, Henan Province, China. Among the participants, boys accounted for 54.3% of the sample, while girls represented 45.7%. Teachers explained the purpose of the survey to the students, and after obtaining informed consent from parents or guardians, coordinated the administration of multiple questionnaires in their classroom settings between September and October 2015. Participation was voluntary, and all data were gathered in compliance with strict confidentiality measures.

### The questionnaire survey

#### The Peer Victimization Questionnaire

The Chinese adaptation of the Olweus Bully/Victim Questionnaire (Olweus, 1993), prepared by Zhang et al. (1999), was used to assess peer victimization. This instrument was widely regarded as a gold standard in the field. The Peer Victimization Questionnaire consisted of 7 items evaluating the type and frequency of bullying experiences encountered by students in the school setting. A total score was calculated by summing responses to all items, each rated on a 5-point scale (0 = never, 1 = once, 2 = twice, 3 = 3~4 times, 4 = 5 or more times). Higher total scores reflected a greater severity of victimization. In this study, the scale demonstrated high internal consistency, with a Cronbach’s alpha of 0.880. The results of the confirmatory factor analysis (CFA) showed suboptimal fit, *χ*^2^_(14)_ = 1038.79, *p* ≤ 0.001, CFI = 0.92, TLI = 0.89, SRMR = 0.04, RMSEA = 0.13. Some fit indices were suboptimal, suggesting the model should be interpreted with caution (Klein, 2012).

#### The Self-esteem Scale

The assessment of self-esteem employed a 10-item instrument derived from the Chinese adaptation of the Rosenberg Self-esteem Scale (Rosenberg and Rosenberg, 1978) by Ji and Yu (1993), which was designed to measure global self-evaluation and self-acceptance. Responses were recorded on a 4-point Likert scale ranging from 1 (“definitely matches”) to 4 (“definitely does not match”), incorporating four reverse-scored items. The final score was derived from the mean of all items, where elevated scores corresponded to higher levels of self-esteem. The scale exhibited adequate internal consistency (*α* = 0.749), and the CFA yielded model fit indices that fell within acceptable thresholds, *χ*^2^_(34)_ = 234.84, *p* < 0.001, CFI = 0.95, TLI = 0.93, SRMR = 0.04, RMSEA = 0.06.

#### The Revised Resilience Scale

The assessment of resilience utilized a revised 13-item scale that synthesized items from the Connor-Davidson Resilience Scale (Connor and Davidson, 2003) and its Chinese adaptation (Hu and Gan, 2008). This instrument measured key components of resilience, including problem-solving, emotional regulation, and psychological toughness. All items were rated on a 4-point Likert scale ranging from 1 (“definitely matches”) to 4 (“definitely does not match”). The overall score was derived from the mean of the 13 items, with elevated scores signifying stronger resilience. The scale exhibited strong internal consistency (*α* = 0.913), and CFA yielded approaching acceptable thresholds, *χ*^2^_(65)_ = 563.26, *p* < 0.001, CFI = 0.89, TLI = 0.87, SRMR = 0.05, RMSEA = 0.09.

#### The Children’s Depression Inventory: Short Form (CDI: S)

The Children’s Depression Inventory: Short form (CDI: S), developed by Kovacs (1992), a tool appropriate for grades 4~9. The scale contained four reverse-scored items, each with three options (scored 0, 1, 2). Higher total scores reflected more severe symptoms. Based on a cut-off score of 7, participants were classified into either a depressive tendency group or a normal group. In this study, the CDI: S had a Cronbach’s alpha of 0.743, and CFA indicated acceptable model fit, *χ*^2^_(35)_ = 312.01, *p* < 0.001, CFI = 0.87, TLI = 0.84, SRMR = 0.04, RMSEA = 0.07.

### Data collection and analysis

We employed SPSS 26.0 and PROCESS v3.0 for all analyses, adopting a significance level of *p* < 0.05. Cases with missing responses on any of the key variables (peer victimization, self-esteem, resilience, depression) were removed listwise. Outliers were identified using boxplot methods and defined as values that exceed ±3 standard deviations. These cases were excluded from analysis. Finally, 337 cases for missing data and 1,190 as outliers were excluded. All continuous predictor variables (peer victimization, self-esteem, resilience) were mean-centered prior to creating interaction terms and run the moderated mediation analysis to reduce multi-collinearity and improve interpretability. Standard regression assumptions (linearity, normality of residuals, homoscedasticity, and independence of errors) were tested and met. No serious violations were detected. We implemented a three-step analytical plan. This began with CFA to validate the scales, all of which showed good model fit and reliability. Next, we examined correlations among gender, peer victimization, depression, resilience, and self-esteem. The final step utilized a moderated mediation analysis (PROCESS Model 59) to test whether the pathway from peer victimization to depression through self-esteem was moderated by resilience.

## Results

### Bivariate correlations

To diagnose potential common method bias, Harman’s single-factor test was conducted. The unrotated factor analysis revealed that two factors had eigenvalues greater than 1, and the first factor accounted for 45.29% of the total variance, which falls below the commonly recommended threshold of 50% (Podsakoff et al., 2003), suggesting that common method bias is not a serious concern in this study.

An elevated prevalence of depressive tendencies (16.20%) and peer victimization (77.20%) was observed in the sample, which reflected the proportion of students who reported experiencing at least one form of peer victimization (i.e., a score ≥1 on any item of the scale). This estimate was consistent with other large-scale studies using similar inclusive criteria (Xu et al., 2020). The interrelationships among the study variables were detailed in Table 1. Correlation analyses indicated that peer victimization was positively associated with depression and inversely associated with resilience, self-esteem, and gender. Furthermore, self-esteem and resilience demonstrated positive mutual correlation and negative correlations with depression. Both constructs were also positively associated with gender. Given that independent-sample *t*-tests confirmed significant gender differences for all key variables (*p* < 0.05), gender was incorporated as a control variable in the subsequent moderated mediation model.

**Table 1:** Descriptive statistics and correlations between five variables.

Variables	1	2	3	4	5
Peer victimization	1				
Self-esteem	−0.331**	1			
Resilience	−0.117**	0.506**	1		
Depression	0.497**	−0.538**	−0.417**	1	
Gender	−0.157**	0.045**	−0.025**	−0.074**	1
Means	1.003	3.041	3.339	3.205	1.460
Standard deviation	0.936	0.491	0.550	3.106	0.498

**p < 0.01.

### Testing for mediation effect

**Figure 1:**
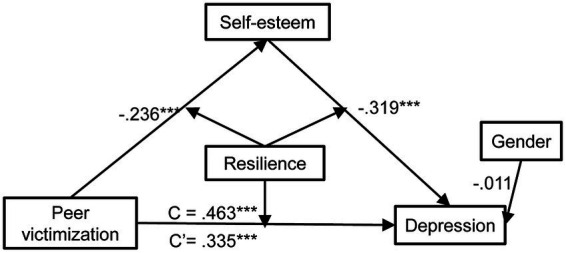
The association between peer victimization and depression was partially mediated by self-esteem. The letter “*c*” represents the overall effect of peer victimization on depression. The letter “*c*′” represents the direct effect of peer victimization on depression. Gender was a controlling variable. ***p < 0.001, **p < 0.01.

Using 5,000 bootstrap samples (*N* = 57,226), a significant indirect effect of peer victimization on depression through self-esteem was found, as the confidence intervals did not include zero (see Figure 1). The total effect of peer victimization on depression was significant and moderate in magnitude (*β* = 0.463, *p* ≤ 0.001, equivalent to Cohen’s *d* ≈ 0.93). When the mediator was included, the direct effect remained significant but decreased to *β* = 0.333 (*p* ≤ 0.001), indicating partial mediation. The indirect effect via self-esteem accounted for 28.08% of the total effect. This proportion, combined with the change in *β*, suggests that self-esteem plays a meaningful but not exclusive mediating role.

### The moderating effect of resilience

We estimated parameters for three regression models to examine these hypotheses. In Model 1, we tested the moderating effects of resilience on the relationship between peer victimization and depression. In Model 2, we evaluated the moderating role of resilience in the relationship between peer victimization and self-esteem. In Model 3, we estimated the moderating effects of resilience on the relations between self-esteem and depression. As Table 2 illustrated, resilience moderated all three pathways of the mediation process.

**Table 2:** Testing the moderating effect of resilience on the relationship between peer victimization and depression via self-esteem.

Variable	Outcome variable: depression			Outcome variable: self-esteem			Outcome variable: depression		
	*β*	*t*	95% CI	*β*	*t*	95% CI	*β*	*t*	95% CI
Gender	0.009	1.296	[−0.004, 0.022]	0.037	5.495***	[0.024, 0.051]	−0.011	−1.925	[−0.023, 0.000]
Peer victimization	0.463	135.665***	[0.456, 0.470]	−0.236	−68.715***	[−0.242, −0.229]	0.335	87.539***	[0.327, 0.342]
Resilience				0.490	134.928***	[0.483, 0.497]	−0.161	−39.751***	[−0.169, −0.153]
Peer victimization * resilience				−0.084	−24.473***	[−0.090, −0.077]	−0.014	−3.408***	[−0.022, −0.006]
Resilience *self-esteem							0.066	17.517***	[0.059, 0.074]
Self-esteem							−0.319	−80.190***	[−0.326, −0.311]
*R* ^2^	0.247			0.324			0.436		
*F*	9408.924***			6728.826***			7306.542***		

***p < 0.01.

**Figure 2:**
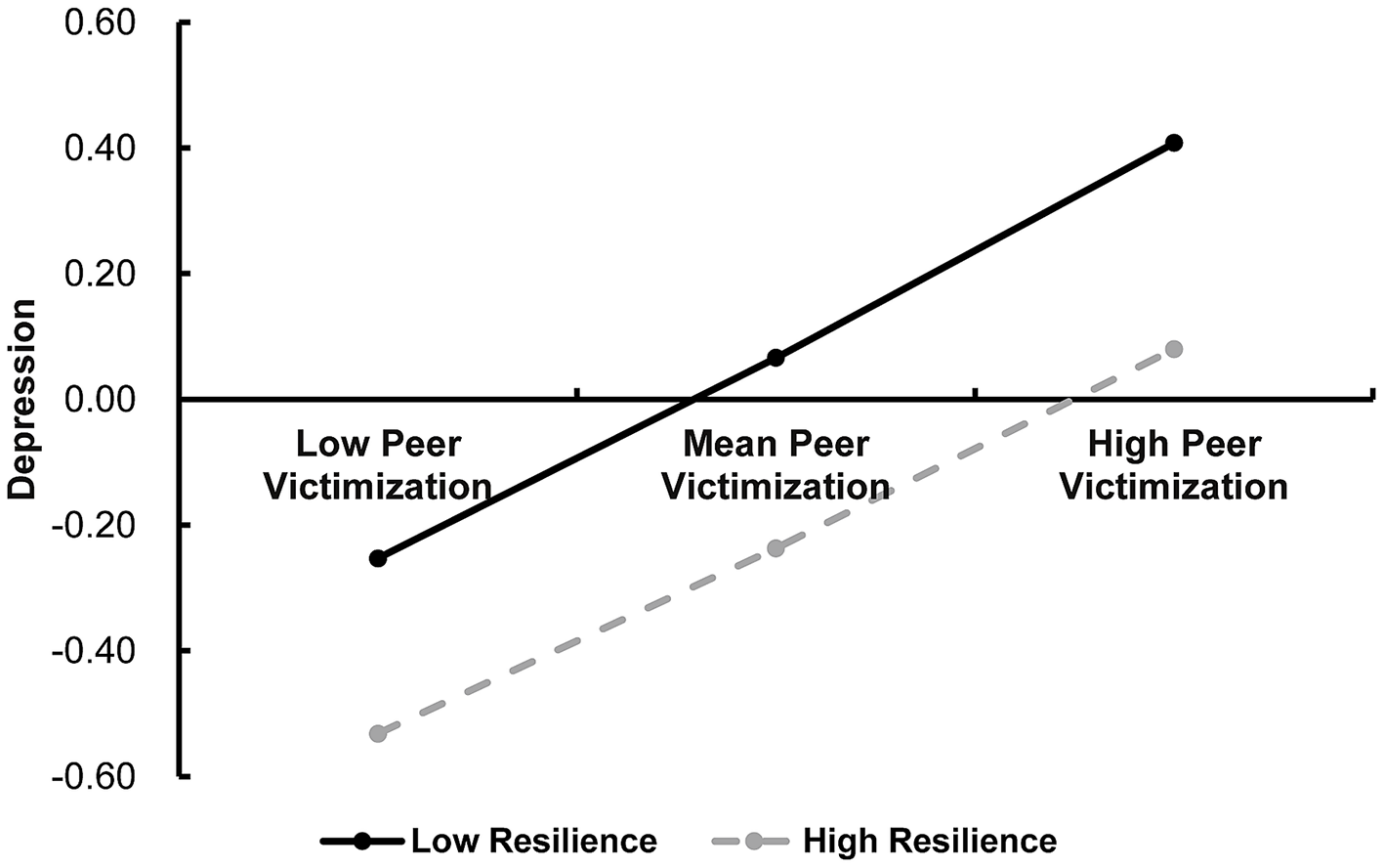
The moderating effect of resilience on the direct relationship between peer victimization and depression (first-stage moderation). Simple slopes show a protective-enhancing pattern: the positive association between peer victimization and depression is weaker for children with high resilience. *X*-axis = peer victimization (standardized), *Y*-axis = depression (standardized). High resilience (+1 SD) and low resilience (−1 SD). This included a legend and noted that slopes were significant at *p* ≤ 0.001.

Model 1 indicated that resilience moderated the association between peer victimization and depression, *β* = −0.014, SE = 0.004, 95% CI = [−0.022, −0.006], indicating that the direct effect of peer victimization on depression was moderated by resilience. Simple slope tests showed that for children with high resilience (1 SD above the mean), peer victimization was positively associated with depression, *β* = 0.322, SE = 0.005, *p* < 0.001. The effect of peer victimization on depression was enhanced with low resilience (1 SD below the mean), *B*_simple_ = 0.348, SE = 0.006, *p* < 0.001, indicating that the buffering effects of peer victimization on depression demonstrated an upward trend with an increase in resilience (Figure 2). Further, the Johnson-Neyman method showed that, in the range of resilience (−2.897, 1.171), the regression coefficients for the effect of peer victimization on depression were significant according to simple slope tests, with all values above zero. Although this interaction effect is statistically significant given the large sample size, its magnitude is small (accounting for approximately 0.02% of unique variance in depression). Nonetheless, the simple slope patterns (see Figure 2) reveal a consistent and theoretically meaningful buffering effect: the protective function of resilience becomes more pronounced as peer victimization increases. Thus, while the interaction term itself is modest, its directional pattern supports a protective-enhancing model.

In Model 2, interactions between peer victimization and resilience had a significant predictive effect on self-esteem, *β* = −0.084, SE = 0.003, 95% CI = [−0.090, −0.077], indicating that the effect of peer victimization on self-esteem was moderated by resilience (First-stage moderation). Simple slope tests showed that for children with high resilience (1 SD above the mean), peer victimization was negatively associated with self-esteem, *B*_simple_ = −0.314, *p* < 0.001. And the effect of peer victimization on self-esteem was still significant and smaller with low resilience (1 SD below the mean), *B*_simple_ = −0.157, *p* < 0.001, indicating that the negative effects of peer victimization on self-esteem demonstrated an upward trend with an increase in resilience. The Johnson-Neyman method showed that, in the range of resilience (−2.897, 1.171), the simple slope tests revealed statistically significant regression coefficients for the effect of peer victimization on self-esteem, with all values less than zero. Although the interaction coefficient (*β* = −0.084) is small in absolute terms, the simple slope analysis shows that the negative effect of peer victimization on self-esteem is nearly twice as large for high-resilience children (*B*_simple_ = −0.314) compared to low-resilience children (*B*_simple_ = −0.157), indicating that resilience amplifies the sensitivity of self-esteem to victimization—a theoretically important pattern even if the interaction term explains limited variance.

In Model 3, interactions between self-esteem and resilience had a significant predictive effect on depression, *β* = 0.066, SE = 0.004, 95% CI = [0.059, 0.074], indicating that the direct effect of self-esteem on depression was moderated by resilience (Second-stage moderation). Simple slope tests also showed that for children with high resilience (1 SD above the mean), self-esteem was negatively associated with depression, *B*_simple_ = −0.256, *p* < 0.001. For low resilience (1 SD below the mean), the association was still significant but stronger, *B*_simple_ = −0.381, *p* < 0.001, indicating that compared with high resilience, the predictive effect of self-esteem on depression was much stronger for children with low resilience. This pattern suggests that the buffering effect of self-esteem against depression is more pronounced when resilience is low, whereas high resilience slightly attenuates this relationship, consistent with a protective-reactive moderation pattern. The Johnson-Neyman method showed that, in the range of resilience (−2.897, 1.171), the regression coefficient values of self-esteem on depression were significant, with every value below zero. The theoretical labels such as protective-enhancing and protective-reactive were used and they clearly match the statistical pattern (Table 3). The interaction between self-esteem and resilience (*β* = 0.066) is small but consistent. Simple slopes reveal that the protective effect of self-esteem on depression is substantially stronger in the low-resilience group (*B*_simple_ = −0.381) than in the high-resilience group (*B*_simple_ = −0.256). This suggests that resilience partly compensates for lower self-esteem, but the effect size cautions against overstating its practical impact. This pattern suggests that the buffering effect of self-esteem against depression is more pronounced when resilience is low, whereas high resilience slightly attenuates this relationship, consistent with a protective-reactive moderation pattern.

**Table 3:** Matching between statistical patterns and theoretical models of resilience.

Path	Interaction sign	Simple slope pattern	Theoretical model
Peer victimization → depression (model 1)	Negative (*β* = −0.014)	High resilience weakens the positive association more strongly at higher victimization levels	Protective-enhancing
Peer victimization → self-esteem (model 2)	Negative (*β* = −0.084)	High resilience shows stronger negative slope; protection diminishes as victimization increases	Protective-reactive
Self-esteem → depression (model 3)	Positive (*β* = 0.066)	Low resilience shows stronger negative slope; resilience attenuates protection	Protective-reactive

## Discussion

The current study aimed to investigate the association between peer victimization and depression using a cross-sectional design. Because causal relationships cannot be inferred from correlational data, all interpretations are presented as patterns of association rather than causal effects. Results demonstrated that peer victimization was significantly associated with depressive symptoms in children, with self-esteem acting as a partial mediator. Moreover, resilience was found to moderate all three pathways within the mediated model.

### The mediating effect of self-esteem

We examined the relationship between peer victimization and depression. Consistent with our hypotheses, peer victimization was positively associated with depressive symptoms in children. This result is consistent with the existing findings (Chou et al., 2020; Hawker and Boulton, 2000; Kaltiala-Heino et al., 1999; Kawabata et al., 2014). Our study also showed that peer victimization was negatively associated with self-esteem, which is in turn related to higher levels of depression. Self-esteem appeared to be an important intermediary factor in the association between peer victimization and depressive symptoms, consistent with a mediation hypothesis that warrants longitudinal testing. The results are in line with existing literature (Copeland et al., 2013; Duru et al., 2019; Klomek et al., 2013; Tsaousis, 2016) and supported by integrated theory of depression (Metalsky et al., 1993). Previous studies have proved that peer evaluation and peer acceptance provide important realtional context when self-esteem is developing (Birkeland et al., 2014; Gorrese and Ruggieri, 2013). Peer victimization usually implies the most dysfunctional relationship with peers, which makes children infer negative evaluations about themselves, such as unacceptable, isolated, unimportant, invaluable, and incompetent, contributing to decreased self-esteem (Gorrese and Ruggieri, 2013). Low self-esteem renders children more prone to making depressogenic inferential attributions about themselves and their interpersonal relationships, which make them infer internal, stable, and highly aversive consequences about bullying and also feel that nothing could change the situation for the better, finally the onset of hopelessness contributing to depressive symptom developed and maintained (Metalsky et al., 1993).

### The moderating effect of resilience

First, this study revealed that, among children with higher levels of resilience, the typically strong association between peer victimization and subsequent depressive symptoms was significantly attenuated. In contrast, for children with lower resilience, greater exposure to victimization consistently predicted higher levels of depression. This pattern aligns with a protective-enhancing model, suggesting that resilience may serve as a protective factor in the association between peer victimization and depression, although causal evidence is needed (Luthar et al., 2000). Children who experience seriously bullying often develop depressogenic inferential style, negative self-evaluation, and a persistent sense of incompetence in coping, which can readily contribute to the sense of hopelessness, thereby resulted in depression. However, high levels of resilience can enhance children’s capacity of emotion regulation during crises, strengthen seeking and utilizing social support effectively, and promote cooperation and solution-oriented thinking (Cohen et al., 2019; Connor and Davidson, 2003), finally preventing the emergence of despair. By navigating and resolving the conflict, victims maybe have learned valuable interpersonal skills. They emerge with increased confidence and a greater sense of well-being, fostering a significant reinforcement of their resilience. Hence, resilience may serve as a protective factor in the association between peer victimization and depression, although causal evidence is needed.

Second, we found a significant negative interaction between peer victimization and resilience on self-esteem (*β* = −0.084, *p* ≤ 0.001). Simple slope analysis revealed that the negative association between peer victimization and self-esteem was stronger for children with high resilience (*B* = −0.314) than for those with low resilience (*B* = −0.157). This pattern indicates that while high-resilience children maintain higher self-esteem under low victimization, their self-esteem declines more steeply as victimization increases. This aligns with a protective-reactive model (Luthar et al., 2000), where a protective factor’s benefit weakens as adversity increases, rather than a purely buffering effect. The results are in line with previous studies (Gong et al., 2020; Overbeek et al., 2010; Palm-Fischbacher and Ehlert, 2014; Tural Hesapcioglu and Yesilova, 2020). The reason maybe that children expose to high levels of peer victimization have been having problems with important personal relationships for long-run (Gorrese and Ruggieri, 2013), such as peer relationship and parent–child relationship, which serve as the foundation for self-esteem development during children (Birkeland et al., 2014; Gorrese and Ruggieri, 2013). Klomek et al. stated that peer victimization has the primarily, central damage on self-esteem, contributing to children’ negative thoughts about themselves and their lives, socially relationships, and their anxiety-related cognitions (Klomek et al., 2008). Thus, notwithstanding high levels of resilience, persistent low self-esteem is a common outcome for teenagers who experience frequent peer victimization (Gorrese and Ruggieri, 2013), and research points to victimization as a principal etiological factor, wherein the internalization of negative self-appraisals due to bullying contributes to the erosion of resilience’s buffering effect (Salmivalli et al., 1999; van Geel et al., 2018).

Third, the interaction between self-esteem and resilience on depression was positive and significant (*β* = 0.066, *p* ≤ 0.001). Simple slope tests showed that the negative association between self-esteem and depression was stronger for children with low resilience (*B* = −0.381) than for those with high resilience (*B* = −0.256). This pattern again reflects a protective-reactive model: when self-esteem is already low, resilience offers limited additional protection against depression (Overbeek et al., 2010; van Geel et al., 2018). Children exhibit stronger resilience are more inclined to form positive self-views and attribute peer victimization to accidental, temporary, and changeable causes (Masten, 2018). Although such cognitive and behavioral resources can mitigate feelings of hopelessness and reduce depressive risk, the protective effect of resilience for these children under low self-esteem (internalization from victimization) is still notably constrained. Our results also revealed that resilience moderated the relationship between self-esteem and depression, but in a pattern consistent with a protective-reactive model rather than a protective-enhancing one. Specifically, for children with low resilience, the negative association between self-esteem and depression was stronger (*β* = −0.381, *p* ≤ 0.001) than for children with high resilience (*β* = −0.256, *p* ≤ 0.001). This indicates that when self-esteem is already low, high resilience offers only limited additional protection against depression. In other words, the protective effect of resilience is most evident when self-esteem is moderately high; however, once peer victimization has been internalized as chronically low self-esteem, resilience cannot fully counteract the depressive risk. This pattern aligns with the protective-reactive model (Luthar et al., 2000; Overbeek et al., 2010; van Geel et al., 2018), wherein a protective factor (resilience) still helps, but its benefit weakens as the level of adversity (here, low self-esteem) increases. Hence, the reason maybe that once internalized as low self-esteem, peer victimization frequently contributed to or exacerbated depression, making it difficult for internal resources such as resilience to exert a protective effect (Overbeek et al., 2010; van Geel et al., 2018). Thus, while resilience is valuable, its limitations should not be overlooked: interventions must directly target the restoration of positive self-evaluation, not solely resilience building.

In summary, three distinct moderation patterns emerged: (a) resilience buffers the direct path from peer victimization to depression (protective-enhancing); (b) resilience exacerbates the negative effect of peer victimization on self-esteem under high victimization (protective-reactive); and (c) resilience attenuates the protective effect of self-esteem on depression (protective-reactive). These findings suggest that resilience is not uniformly beneficial. Its protective function depends critically on the specific pathway and the level of adversity.

In conclusion, this study confirmed that peer victimization has both direct and indirect effects on depression. Self-esteem was an important mediating factor between peer victimization and depression, and resilience moderated the mediation process. Our results emphasized that peer victimization was a major risk factor that uniquely contributed to lower self-esteem and later depression. Resilience can exert its full protective effect as long as the experience of victimization have not been internalized into stable low self-esteem (Overbeek et al., 2010; van Geel et al., 2018). Our results lend support to the integrated theory of depression (Metalsky et al., 1993), which emphasizes that depression cannot be attributed to a single cause but arises from dynamic interactions across multiple systems. It is important to emphasize that the present study employed a cross-sectional design; therefore, the observed relationships among peer victimization, self-esteem, resilience, and depression are correlational in nature. Although our moderated mediation model was grounded in established theoretical frameworks—such as the integrated theory of depression (Metalsky et al., 1993) and resilience models (Luthar et al., 2000)—which imply directional pathways, the current data cannot support causal conclusions. For instance, while we found that peer victimization statistically predicted lower self-esteem and higher depression, alternative directional interpretations (e.g., depressed children may be more vulnerable to peer victimization) or bidirectional effects are equally plausible based on cross-sectional evidence (van Geel et al., 2018).

Effective interventions for depression might consider both the external environment—by providing support—and internal factors, such as resilience and self-esteem. Firstly, schools may consider establishing clear regulations and implementing anti-bullying programs. Bullies might benefit from consistent consequences. Bullies must face stricter consequences to prevent them from gaining a sense of superiority through such acts. Ultimately, bullying can only be truly stopped when bullies become consciously aware of the harm they cause to others—and to themselves. Secondly, this study revealed that resilience has sufficient protection on depression related to peer victimization. Moreover, children reporting low resilience vs. high resilience always has lower self-esteem, regardless of the level of peer victimization. Also, individuals with low resilience vs. high resilience consistently exhibit higher levels of depression, irrespective of the level of self-esteem, implies that low resilience may be an essential risk factor for both low self-esteem and depression. Hence, intervention efforts for parents and teachers might consider enhancing key competencies of resilience, such as emotional management, effective problem-solving, the ability to draw on social support, focused attention, toughness, and a strong sense of self-efficacy (Moore and Woodcock, 2017). Third, the current study demonstrated that self-esteem served as an important mediator mechanism between peer victimization and depression. Extremely destructive relationships are internalized as children’s low self-esteem, which in turn leading to the onset of depression and attenuated the protective effect of resilience. Once stable low self-esteem develops following bullying incidents, children may become trapped in a cycle of repeated victimization and depression. So, teachers and parents might consider offering more attention and encouraging children to develop more positive self-evaluations, pending further evidence from causal designs, such as the feeling of security, meaningful, self-efficacy, self-acceptance, and coping capacity, to reduce their tendency of maladaptation.

### Limitation and future direction

Several limitations must be considered when interpreting the results of this study. First, the current study employed a cross-sectional design, which fundamentally restricts causal inference. While our proposed mediation model implies a directional process (peer victimization → self-esteem → depression), cross-sectional data can only confirm statistical associations, not causal ordering. Alternative models—such as depression leading to increased victimization risk or reciprocal effects over time—are equally consistent with cross-sectional findings (van Geel et al., 2018). Therefore, all uses of terms such as “predictor”, “effect”, and “indirect effect” in this paper refer to statistical predictions based on regression models, not to causal relationships. Therefore, future research should employ longitudinal panel designs (e.g., cross-lagged models) or experimental interventions to test the temporal precedence and causal assumptions of the present model. Second, this study relied exclusively on children’s self-report measures, which introduces the risk of common method variance potentially inflating the observed relationships. Self-reports of sensitive topics such as peer victimization and depressive symptoms may also be subject to recall inaccuracies, social desirability bias, and momentary mood effects. To address these limitations, future research should incorporate multiple informants (e.g., teacher ratings of peer victimization, peer nominations, parent reports of child adjustment) and, where possible, observational or clinical assessments. Multi-method designs would help disentangle method-specific variance from substantive relationships. Third, the findings are based exclusively on a sample of fourth-grade students from urban public schools in Zhengzhou, China. This specificity limits generalizability to other developmental stages (e.g., adolescents, where peer victimization patterns and self-esteem processes may differ), rural populations (where social dynamics and resources vary), and other cultural contexts (where the meaning and expression of self-esteem, resilience, and depression may differ). Future research should test the proposed moderated mediation model in diverse samples, including adolescents, clinical populations, rural communities, and cross-national settings, to establish the boundary conditions of our findings. Fourth, methodologically, school-level clustering is a key consideration. Although our individual-level analyses were appropriate given the focus on individual psychological processes, future studies should use multilevel modeling to account for possible clustering effects. Finally, the measurement model fit for some scales, particularly peer victimization (RMSEA = 0.13), was less than ideal. While large samples can inflate *χ*^2^ values, the RMSEA exceeded conventional acceptable limits (Hu and Bentler, 1999), indicating possible problems with the adapted Olweus scale’s factor structure in this Chinese sample of fourth graders. Future studies should use stronger psychometric measures or multi-informant methods (e.g., peer nominations, teacher reports) to assess victimization more reliably.

## Data Availability

The raw data supporting the conclusions of this article will be made available by the authors, without undue reservation.
